# Fate of sloughed biomass in integrated fixed-film systems

**DOI:** 10.1371/journal.pone.0262603

**Published:** 2022-01-21

**Authors:** Hussain Aqeel, Steven N. Liss

**Affiliations:** 1 Department of Chemistry and Biology, Ryerson University, Toronto, Ontario, Canada; 2 School of Environmental Studies, Queen’s University, Kingston, Ontario, Canada; 3 Department of Microbiology, Stellenbosch University, Stellenbosch, South Africa; South China University of Technology, CHINA

## Abstract

Fate of biofilm sloughing was assessed in a laboratory-scale (LS) integrated fixed-film sequencing batch reactor (IF-SBR) treating synthetic wastewater and in a full-scale (FS) integrated fixed-film activated sludge (IFAS) system treating municipal wastewater. It was observed that the properties of biofilms and flocs, including sludge volume index (SVI), mixed liquor suspended solids (MLSS), effluent suspended solids (ESS), relative hydrophobicity, and composition of extracellular polymeric substance (EPS) were associated with biofilm sloughing and formation of large granular flocs in the LS IF–SBR. In the FS IFAS system, the changes were studied at the molecular level. For example, the extracted EPS content results (the protein to polysaccharide ratio decreased in the flocs and increased in the biofilms, with biofilm sloughing) were complemented with the confocal laser scanning microscopy (CLSM) coupled with molecular specific staining. CLSM analyses revealed that micro-colonies rich in polysaccharides readily sloughed from the carriers. Live-dead staining revealed areas of the biofilm where the viability of biomass was a contributing factor associated with areas of the biofilm susceptible to sloughing. 16S rRNA gene sequencing (Illumina) of FS IFAS samples revealed greater diversity (α-diversity) in biofilms compared to flocs. Biofilm sloughing resulted in a decrease in diversity in biofilms and a corresponding increase in the flocs during sloughing. Microbial population dynamics revealed that bacteria known for denitrification (for example, *Comamonadaceae*) detached from the biofilms during sloughing, readily associated with the suspended biomass, and were retained in the bioreactors.

## 1 Introduction

The first full-scale installation of the IFAS technology was reported in the mid-1990s [1]. The application of the IFAS technology is an easy option to upgrade the existing conventional wastewater treatment facilities [2–4]. Several case studies have shown year-round stability and improvement in capacity and efficiency of BNR when a conventional activated sludge system was upgraded to an IFAS system [5–7]. The hybrid nature of IFAS takes advantage of the differential features of biofilms and flocs that contribute differently to biological nutrient removal (BNR) [8, 9]. For example, the relative abundance of denitrifying bacteria is higher in biofilms; whereas, the abundance of nitrifying bacteria is relatively higher in the flocs of a hybrid IF-SBR [10].

Integrated fixed-film activated sludge (IFAS) systems are hybrid bioreactors, employing both attached biomass (biofilms developed on carrier media) and suspended-growth biomass (flocs) for BNR. The characteristics of biofilms and flocs depend on the size of the microbial aggregate and microbial community composition. The resistance to substrate transport is relatively less in small flocs (smaller than 0.2 mm) compared to the large flocs (larger than 0.2 mm) and mature biofilms. The stratified structures (with oxic, anoxic, and anaerobic zones) are formed in the large microbial aggregates (to support the growth of diverse microbial community) that can facilitate the simultaneous removal of nitrogen, phosphorus, and organic loading from wastewater [3, 11, 12]. The diverse redox conditions in the IFAS system facilitate the growth of a diverse microbial community in the hybrid system. The combination of biofilms and flocs in the IFAS system shows a synergistic effect for BNR. In one study, the nitrogen removal rate of the MBBR system was found to increase three-fold in an integrated system [6].

Biofilm development is a dynamic process that starts with initial microbial adhesion on a surface followed by maturation and then biofilm dispersion [13]. The processes of biomass detachment from biofilm include grazing by eukaryotes and abrasion due to physical collision, in addition to erosion and sloughing [14]. Erosion (due to external shear forces) can lead to the dispersion of small particles leaving behind a smooth biofilm surface [15]. Whereas detachment of large particles, or sloughing events, usually due to internal instability, results in uneven and rough surfaces [15]. Several physical, chemical, and biological factors have been recognized to induce biofilm sloughing. Biofilm sloughing is considered an integral part of biofilm development [16, 17]. In fixed-film wastewater treatment systems, biofilms grow under continuous hydrodynamic and shear pressures facilitating the mass transport to the core and forming compact biofilms [18]. Environmental biofilms are organized mixed microbial aggregates. In response to a stressor, microbes in conditions unfavorable for growth disperse from the biofilm. The initial dispersion of biofilm may follow regrowth and reorganization of the biofilm based on new operational and nutritional conditions, or result in further biofilm disintegration [11, 14].

This paper reports on the fate of sloughed biomass in integrated fixed-film systems. We monitored the biofilm characteristics associated with sloughing events using microscopic observations and microbial community analyses. To our knowledge, the sloughing has not been well documented or investigated in the hybrid bioreactors. We discuss that the retention of sloughed biomass in a hybrid system contributes to microbial community dynamics of the suspended biomass. The examination of biofilm sloughing is of increasing importance given the role of biofilms and hybrid systems in the future of BNR and other advances in biological wastewater treatment.

## 2 Materials and methods

### 2.1 Experimental setup

In this study, biofilm sloughing in the LS IF-SBRs, and FS IFAS system was studied. The use of SBR in a laboratory (bench-scale studies) as a basis for simulating biological wastewater treatment systems, including phenomena associated with activated sludge processes, is well established, e.g. Liao et al. [19]. The SBR configuration permits the return of settled biomass without a requirement for secondary clarification, and the conditions throughout the react cycle are like plug-flow conditions. The changes in properties of biofilms and flocs that were found associated with biofilm sloughing in LS IF-SBR were also studied in the FS IFAS system. Furthermore, the FS IFAS samples were used to reveal the changes in extracellular polymeric substance (EPS) composition at the molecular level, and microbial community composition using 16S rRNA gene sequencing.

We have extensively employed laboratory-scale bioreactors, including configurations incorporating integrated fixed-film systems, and observations of full-scale systems [8, 11, 20]. It was observed that there was no periodicity in biofilm sloughing, and it happens spontaneously. However, changes in the properties of the biofilms and flocs were observed. Therefore, visual and microscopic observations were used to identify sloughing events.

#### 2.1.1 IF-SBR setup

**Fig 1:**
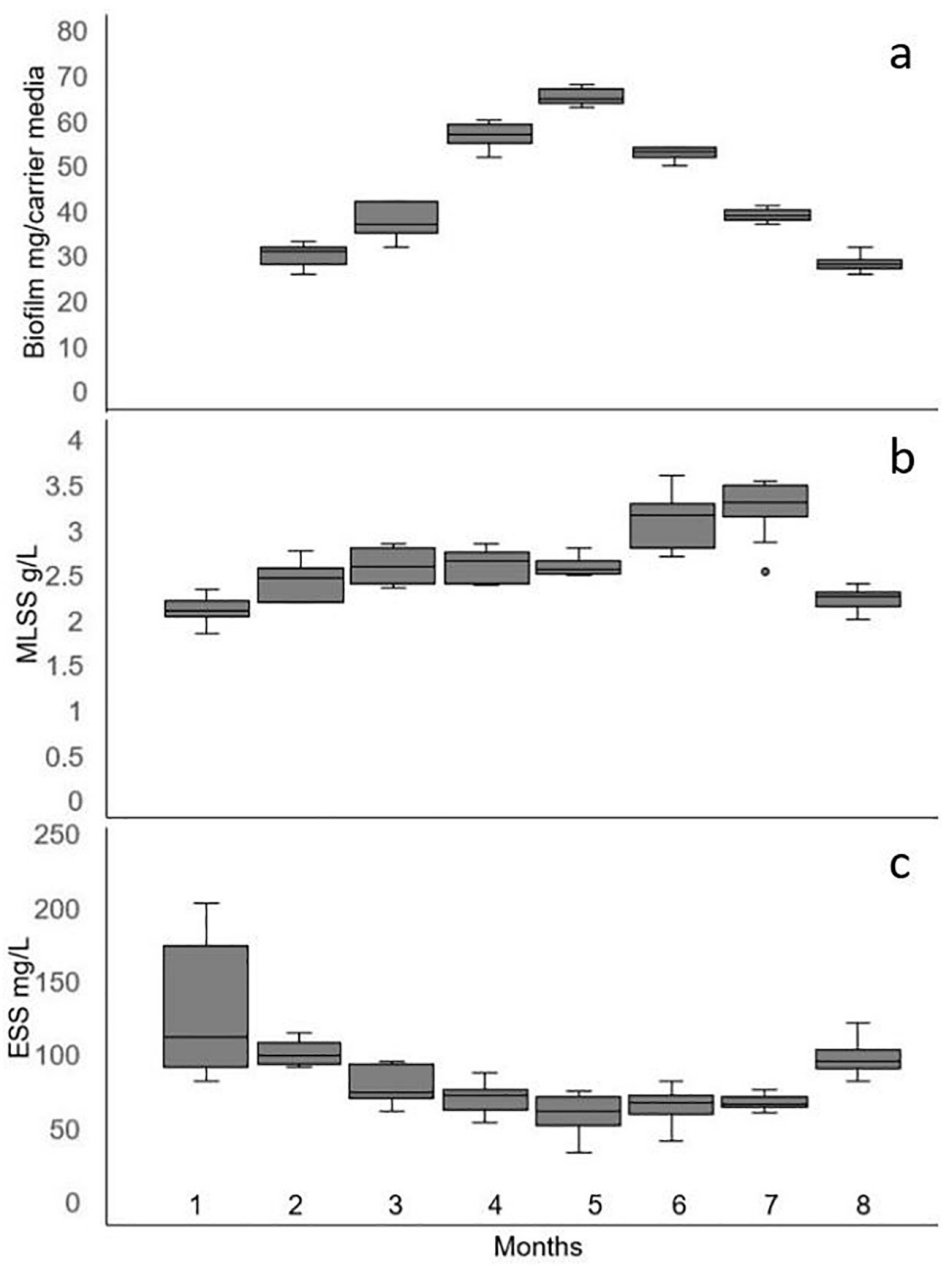
The box plot showing distribution of biomass content in the LS IF SBR (a) attached biomass on the carrier media, (b) MLSS, and (c) ESS. The X-axis shows the months of bioreactor operation.

Two LS IF-SBR with an effective working volume of two liters each were operated for eight months. The LS IF-SBR were seeded with activated sludge from a municipal wastewater treatment plant (Cataraqui Bay wastewater treatment plant, Kingston, Ontario, Canada). The bioreactors were supplemented with biofilm carriers composed of polyethylene, two centimeters in size, and 139 m^2^/m^3^ surface area [8]. The biofilm carriers in the LS IF-SBR were filled to 30% (volume/ volume). The operating conditions of the LS IF-SBR are described in Table 1 and Fig 1 and are similar to the SBR systems described previously in several studies examining flocculation, granulation, and biofilm formation [7, 19, 21]. The LS bioreactors were operated with six hours of sequencing batch cycles that fill for 15 minutes, react for 315 minutes, settle for 15 minutes, and then withdraw for 15 minutes. The IF-SBR were fed with glucose-based synthetic feed consisting of a chemical oxygen demand (COD)/ nitrogen/ phosphorus ratio of 100/ 5/ 1. The macro and micronutrient composition for synthetic feed were described previously [21]. The synthetic feed composition (in mg/L) was glucose (300), NH_4_Cl (57.3), KH_2_PO_4_ (13.2), MgSO_4_ (2.48), FeSO_4_·7H_2_O (2.49), NaMoO_4_·2H_2_O (1.26), MnSO_4_·4H_2_O (0.31), CuSO_4_ (0.25), ZnSO_4_·7H_2_O (0.44), NaCl (0.25), CaSO_4_·2H_2_O (0.43), and CoCl_2_·6H_2_O (0.41). The synthetic feed was prepared daily and stored at 4°C until fed into the bioreactors. The pH of the feed was adjusted to 7.2 ±0.2 with a 1 M NaOH solution. LS IF-SBR were equipped with feeding and effluent pumps (Cole Parmer model 7520–35), air pumps (Optima LR-91926) connected with a diffused air stone for aeration, and a magnetic stirrer to mix the biomass. The air stone was kept at 0.4 L level for aeration and to minimize interference to the magnetic stirrer.

**Table 1:** LS IF-SBR operating conditions and performance.

Parameters	LS-IF-SBR
SRT (days)	9±1
HRT (hours)	8
Temperature (°C)	20±2
Organic substrate (mg/L)	300
COD removal (%)	99±1
*SVI_30_ (ml/g MLSS)	341±7
*SVI_30_/ SVI_10_	0.8
Reactor operating cycle	
Fill, settle and withdraw phases (min)	15 each
Reaction phase (min)	315

* The settling properties reflect conditions when large granular floc formation was observed. This occurred following four months of operation of the LS IF SBR.

The LS IF-SBR were operated at room temperature (20 ±2°C), with a hydraulic retention time (HRT) of eight hours [21]. The solid retention time of six days was controlled for the first 45 days of operation, and then it was increased to nine days in LS IF-SBR. The mixed liquor suspended solids (MLSS) and effluent suspended solids (ESS) were measured following standard methods [22] to calculate Solid retention time (SRT). The biomass was wasted during the start of the SBR cycle every day to maintain the intended SRT (based on suspended biomass). However, the SRT in the hybrid bioreactors was relatively higher because the biofilms attached to the carrier media usually have a higher retention time. Relative hydrophobicity was measured using a hydrocarbon-binding assay [23]. Sludge volume index (SVI) was measured as described previously [19]. COD was measured by photometric analysis using COD reagent vials, following the manufacturer’s protocol (Canadawide Scientific). Dissolved oxygen (DO) concentrations and pH were measured with DO/pH meter (Oakton).

#### 2.1.2 FS IFAS system setup

**Fig 2:**
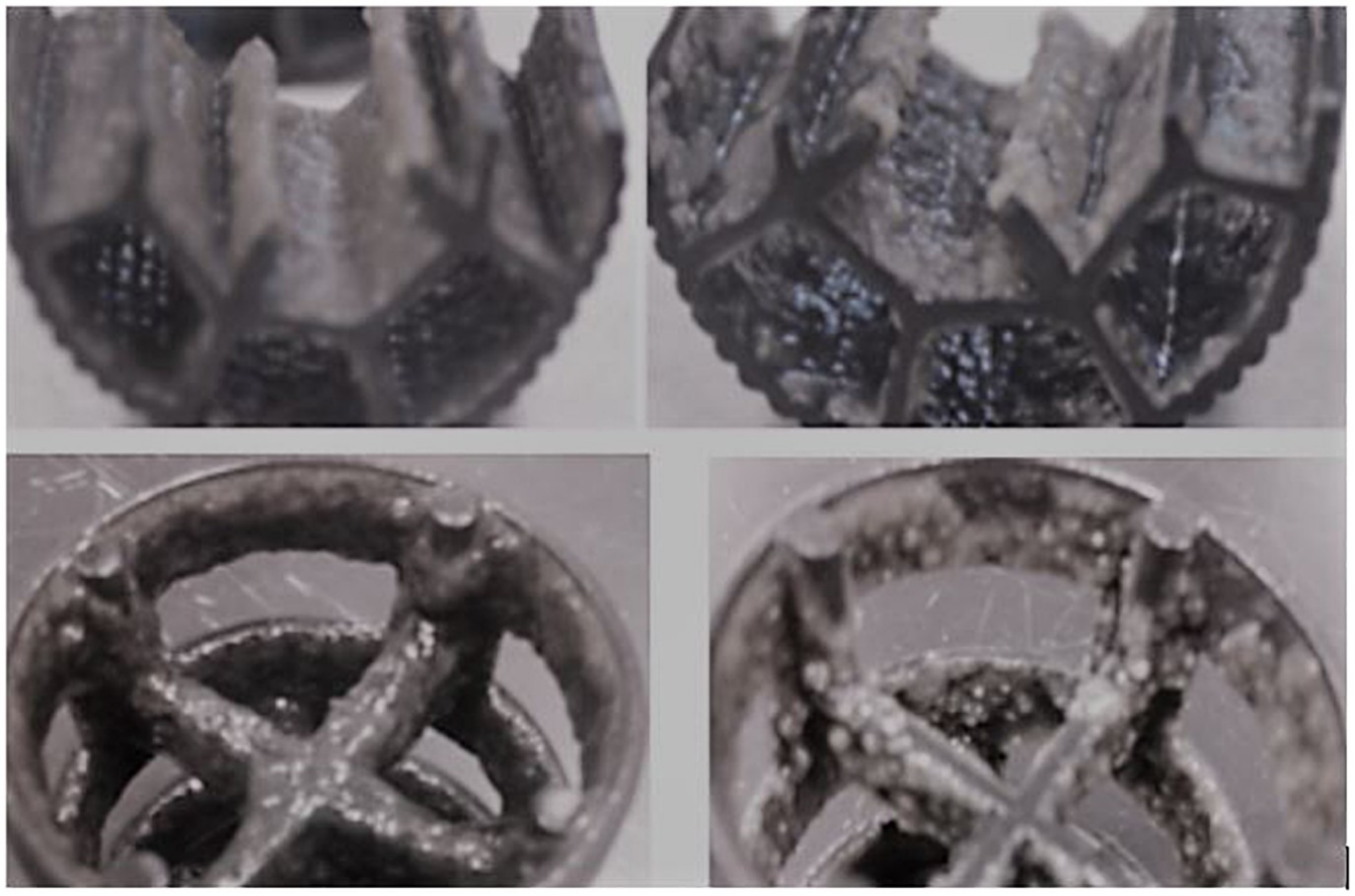
Plastic carrier media of LS IF SBR (upper row) and FS IFAS (lower row) systems with intact (left column) and sloughed biofilms (right column).

FS IFAS system (municipal wastewater treatment plant, Peterborough, Ontario, Canada) was upgraded from a conventional activated sludge system by adding carrier media in the aeration tank. The carrier media was composed of polyethylene with a protected surface area of 210 m^2^/m^3^. The retrofitting of the conventional wastewater treatment plant improved treatment capacity and the quality of effluent to meet the standards for minimum biosolids and nutrients. The FS IFAS setup, operating conditions, and performance of biological nutrient removal were published elsewhere [5]. Biomass samples were collected randomly from the aeration tank of the FS IFAS system (Peterborough, Ontario, Canada), treating municipal wastewater. The samples consisted of carrier media and flocs retrieved before, during, and after biofilm sloughing from the FS IFAS system. The term “before sloughing” means the samples were collected during stable operating conditions where biofilm was observed to be intact, and there were no changes in suspended biomass. The term “during sloughing” the sloughed biofilms on the carrier media and increased suspended biomass were observed. The term “after sloughing” means when the regrowth of biofilm was observed, and there were no further changes in the suspended biomass. The suspended solids of the FS IFAS aeration tank were measured five to seven days a week. The MLSS of the aeration tank ranged from 1.2 g/L to 2.2 g/L. The HRT and SRT of the bioreactors were four hours and six days, respectively. The change in MLSS and visual observation of the carrier media was used as a screening step to identify the biofilm sloughing. The properties of the FS IFAS biofilms and flocs are described in Table 2. Verification and differentiation of biofilm and floc samples were based on microscopic observations of the biofilms on carrier media (Fig 2) and flocs throughout the sampling period.

**Table 2:** Properties of FS IFAS biofilms and flocs associated with biofilm sloughing.

	Before sloughing	During sloughing	After sloughing
SVI (ml/g MLSS)	118	108	112
Floc hydrophobicity (%)	31.1±0.1	26.7±5	52.9±0.4
Biofilm hydrophobicity (%)	24.3±1	23.2±2	71.5±0.3
Biofilm per carrier media (mg)	134	96	133
EPS			
Floc protein (mg/g MLLS)	111±3	113±2	156±5
Biofilm protein (mg/g MLLS)	94±2	116±4	133±2
Floc polysaccharide (mg/g MLLS)	18±1	68±2	17±1
Biofilm polysaccharide (mg/g MLLS)	20±1	27±1	52±2
Floc PN: PS	5.6	1.7	9.2
Biofilm PN: PS	4.7	4.3	3.0

### 2.2 Structural properties of biofilms and flocs

Biofilm and floc samples were collected from LS IF–SBR and FS IFAS systems that were processed for further analyses. Floc size was determined by observing samples by phase-contrast microscopy (Olympus model BH2-RFCA). The biofilm thickness was measured with confocal laser scanning microscopy (CLSM). However, it was observed that when biofilm thickness increased to more than 200 μm, CLSM was unable to measure the thickness of biofilm. Therefore, the dry biomass of biofilm per carrier media was measured by weighing the carrier media before and after cleaning the carrier media [8, 20]. Briefly, biofilm carrier media samples were gently rinsed with deionized water to remove the flocs from the biofilm surface. Then biofilm carrier media were placed in an oven at 100°C for one hour. The dry biomass on carrier media was weighted, then washed twice with 0.1 M NaOH solution (at 80°C) with vigorous shaking. After washing, the carrier media were rinsed with deionized water and dried in an oven at 100°C for one hour [10, 20]. The clean and dry carrier media were weighted to determine the dry biomass per carrier medium.

#### 2.2.1 Extraction and quantification of extracellular polymeric substance

The extracellular polymeric substance (EPS) from the biofilms and flocs was extracted using a cation exchange resin (CER) in the sodium form (Dowex^®^, Sigma-Aldrich, Canada), as described elsewhere [8, 24]. The biomass samples were washed thrice with deionized water and centrifuged at 9,000 g for 10 min at 4°C. The pellet was resuspended in a pH 7.0 phosphate saline buffer (PSB) (2 mM Na_3_PO_4_, 4 mM NaH_2_PO_4_, 9 mM NaCl and 1 mM KCl) to wash the biomass. The pellet was resuspended in 1 ml PSB to form a suspension of biomass of 10 g/ L. The biomass suspensions were vortexed for 60 min at 4°C with the CER. The sample/CER was centrifuged at 9000 g for 10 min 4°C, and the supernatant was collected to quantify the EPS contents. The concentration of protein and polysaccharides in the extracted EPS was measured as described previously [8]. Total protein content in EPS was quantified using bovine serum albumin as standard and following the phenol-sulfuric acid method [25]. The total polysaccharide was quantified using glucose as a standard [26].

#### 2.2.2 Confocal laser scanning microscopy

The floc samples were prepared for staining, as described previously [21, 27]. The biofilm samples were gently rinsed with deionized water to remove unbound particles from the biofilm surface. Two to three plastic carrier media were sacrificed and sliced with a surgical blade into several small one-centimeter pieces of carriers with intact biofilm. The small pieces of carriers with biofilms were stained in a Petri dish (2.5 cm diameter) that were placed on a shaker (50 rpm) at room temperature in the dark for 30 minutes. After staining, samples were gently rinsed twice with deionized water to remove excess stain. The glass slides were mounted with double-sided sticky tapes to stabilize the small pieces of carrier media with biofilms for microscopy. Thick biofilms were placed in cryo-sectioning molds, covered with cryo-sectioning solution, and stored overnight at -20°C. The frozen biofilms were sliced into 20 μm cryo-sections. The biofilm slices were directly collected and stained on glass slides with 300 μL stain cocktails. The slides were kept in a dark and humid chamber for 30 minutes and then gently rinsed with deionized water to remove the unbound stain.

Combinations of multiple fluorescent stains with different spectral characteristics were used to probe the distribution of EPS and cells in biofilms and flocs [21, 27]. A cocktail of three stains was prepared with FITC, concanavalin A, and calcofluor white to probe localization of the protein, α-polysaccharide, and β-polysaccharide, respectively (S1 Table). A combination of SYTO 9 and propidium iodide stains was used to estimate live and dead cells, respectively.

The stained samples were analyzed using 10–20 microscopic fields. All 2D and Z stack images were captured using a 10X magnification lens, using ZEN 2009 software (Zeiss LSM 710). ZEN 2012 (Zeiss) software was used for image analysis, including mean intensity measurements [8, 27]. The excitation and emission settings used for all probes are presented in S1 Table.

### 2.3 DNA extraction and amplicon sequencing

Genomic DNA was extracted from biofilm and floc samples collected from the FS IFAS system (described above), using the PowerSoil DNA Isolation Kit (MoBio Laboratories Inc.). The genomic DNA from the FS IFAS samples was extracted in duplicate from each biofilm and flocs sample. The duplicate samples were pooled together to make a representative sample from biofilms and flocs. The composition of the bacterial population in biofilms and flocs was assessed using 16S rRNA gene sequencing following a previously published method [28]. The V3-V4 region of the 16S rRNA gene was amplified using primers 341F (CCTACGGGNGGCWGCAG) and 805R (GACTACHVGGGTATCTAATCC), with sample-specific barcodes. The PCR product was sequenced using Illumina MiSeq. The Illumina sequencing was performed at Genome Quebec. Taxonomic classification, α (observed species), and β (weighted UniFrac) diversity metrics were performed using QIIME software suit. The sequences were assigned using the RDP classifier with a modified Greengenes database [29–31]. All sequence data associated with this study were deposited in the European Nucleotide Archive under study ID PRJEB15458.

## 3 Results

### 3.1 Reactor operation and biomass growth

The LS IF-SBR were seeded with activated sludge from a conventional municipal wastewater treatment plant (Cataraqui Bay wastewater treatment plant, Kingston, Ontario, Canada). The seed biomass was composed of small and loosely bound flocs with filamentous bacteria and eukaryotic organisms (nematodes, rotifers, amoeba, and ciliates). Polyethylene carriers (30% fill volume) were added in the bioreactor to support the development of biofilms. The protective surface area of each carrier media was 139 m^2^/m^3^ [8]. During LS IF-SBR operation, chemical oxygen demand (COD) removal was 99 ±1%, and dissolved oxygen (DO) concentration was maintained at 3–5 mg/L.

Solid retention time (SRT) of six days was maintained during the first 45 days of IF-SBR operation. The effluent suspended solids (ESS) were observed to be relatively higher (100–200 mg/L) during the initial stabilization phase. After the first 45 days of SBR operation the biomass retention increased and ESS decreased. Therefore, the SRT was increased to nine days (Fig 1). The initial setup and transition to a higher SRT and observations concerning ESS were similar to an earlier study [19].

Mature biofilm was developed within the first two months of the IF-SBR operation that gradually gained mass up to the fifth month. The dry weight of the attached biomass growth on carrier media in the LS IF-SBR system is presented in Fig 1. Patches of biofilm appeared within the first two months of operation (26–36 mg biomass/ carrier media). A confluent biofilm was developed on the carrier media following the first three months of the IF-SBR operation. The biomass attachment increased during the fourth month of the IF-SBR operation that was observed with an increase in dry weight of biofilms on carrier media. However, biofilm sloughing was also observed, which was determined by microscopic observation of biofilms and the formation of large and compact flocs. The maximum attached biomass per carrier media (63–67 mg) was observed after five months of IF-SBR operation, which decreased gradually following a major biofilm sloughing event (Fig 1). In the FS IFAS system, the dry weight of the developed biofilm per carrier media (131–137 mg) decreased during biofilm sloughing (96 ±2 mg) and subsequently recovered (133 ±5 mg) following biofilm sloughing (Table 2).

The dry weight of mixed liquor suspended solids (MLSS) during the initial bioreactor setup at SRT of six days was 1.7–2.3 g/L, which increased to 2.4–2.8 g/L when SRT was increased to nine days (3–5 months of SBR operation). A major biofilm sloughing event on day 202 initially increased MLSS (2.5–3.5 g/L) followed by a decrease in MLSS (2.0–2.4 g/L) when the dry weight of the attached biofilm decreased to 30 mg per carrier media (Fig 1). Visible changes in the appearance of the biofilms on the carrier media were observed. Microscopy observations revealed that the biofilm surface was relatively smooth before sloughing. However, peaks and valleys were observed on biofilm surfaces after sloughing, indicating patches of biomass were detached from the surface of the biofilm (Fig 2). The range of flocs size was 50–150 μm during the first four months that increased to 50–500 μm after four months of the IF-SBR operation.

The formation of large and compact flocs resulted in improved settling properties of the suspended biomass with an SVI of as low as 34 ml per gram MLSS. An increase in MLSS indicates the formation of granular sludge. The ratio SVI_30_/ SVI_10_ is an important indicator of fast settling properties. A ratio of SVI_30_/ SVI_10_ closer to one indicates the completeness of granulation. Many studies have shown that a ratio of one is usually achieved during granulation; however, some granulation studies have been reported with suboptimal values of 0.8–0.9 [21, 32, 33]. The ratio SVI_30_/ SVI_10_ of the granular flocs in the present study was 0.8 (Table 1) in IF-SBR. The SVI_30_/ SVI_10_ value below one indicates the presence of flocs with the granular sludge. The co-occurrence of small flocs with granular sludge was also confirmed by microscopic observation in this study.

### 3.2 Properties of biofilms and flocs during sloughing

Initially, the biofilm sloughing was observed in the LS bioreactors that were operated for another study. The analyses of the biofilms and flocs properties show association with the sloughing. Later, the properties of FS-IFAS biofilms and flocs were studied to observe if a similar pattern exist in the FS system. Additionally, the FS-IFAS samples were used for further in-depth molecular analyses, such as confocal microscopy and 16R rRNA gene sequencing analyses.

#### 3.2.1 Distribution of EPS and live/dead cells

**Fig 3:**
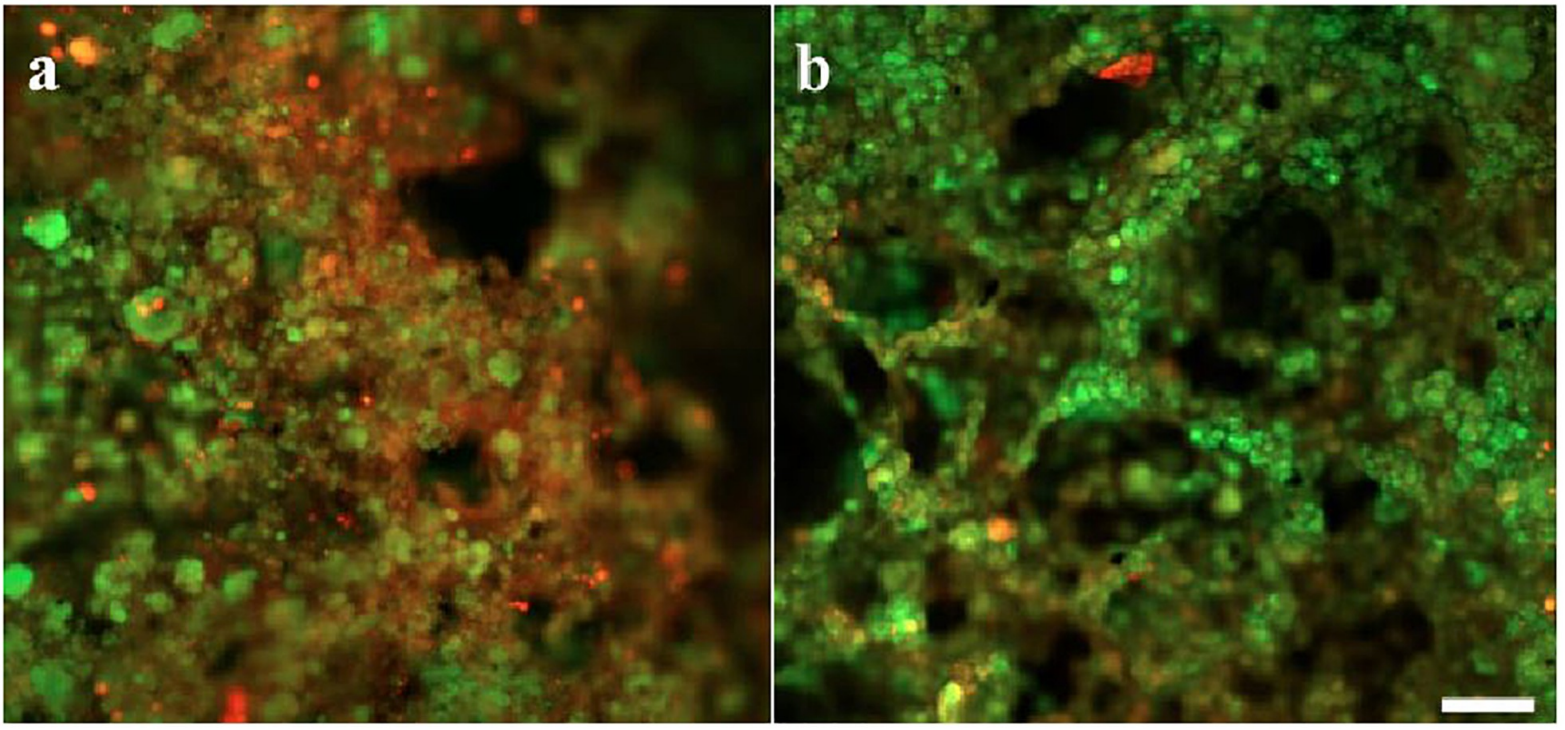
Distribution of live (green) and dead cells (red) in FS IFAS biofilms (a) intact and (b) sloughed biofilm. Scale = 200 μm.

**Fig 4:**
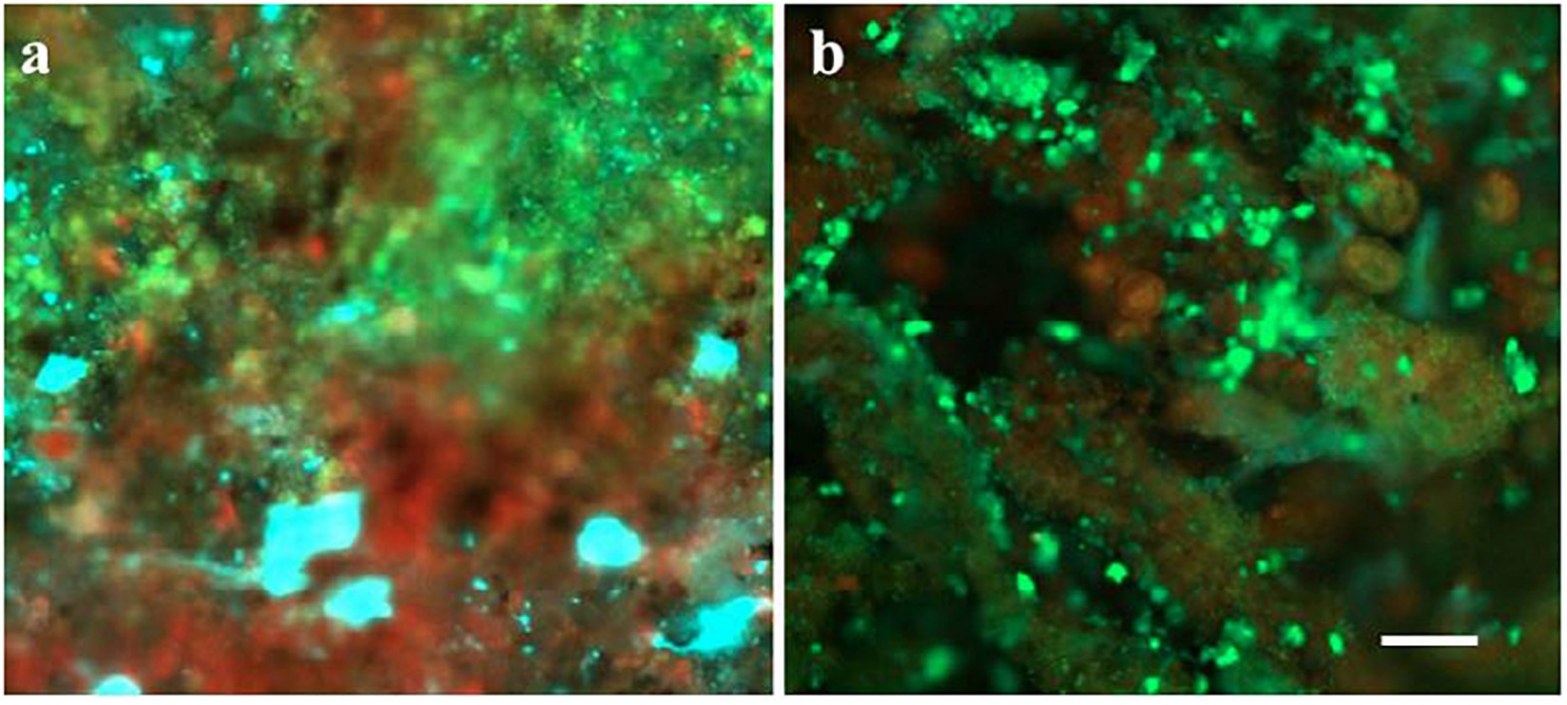
Distribution of protein (green), alpha polysaccharides (red), and polymers with β structures (cyan) in FS IFAS biofilms, (a) intact, and (b) sloughed biofilm. Scale = 200 μm.

**Fig 5:**
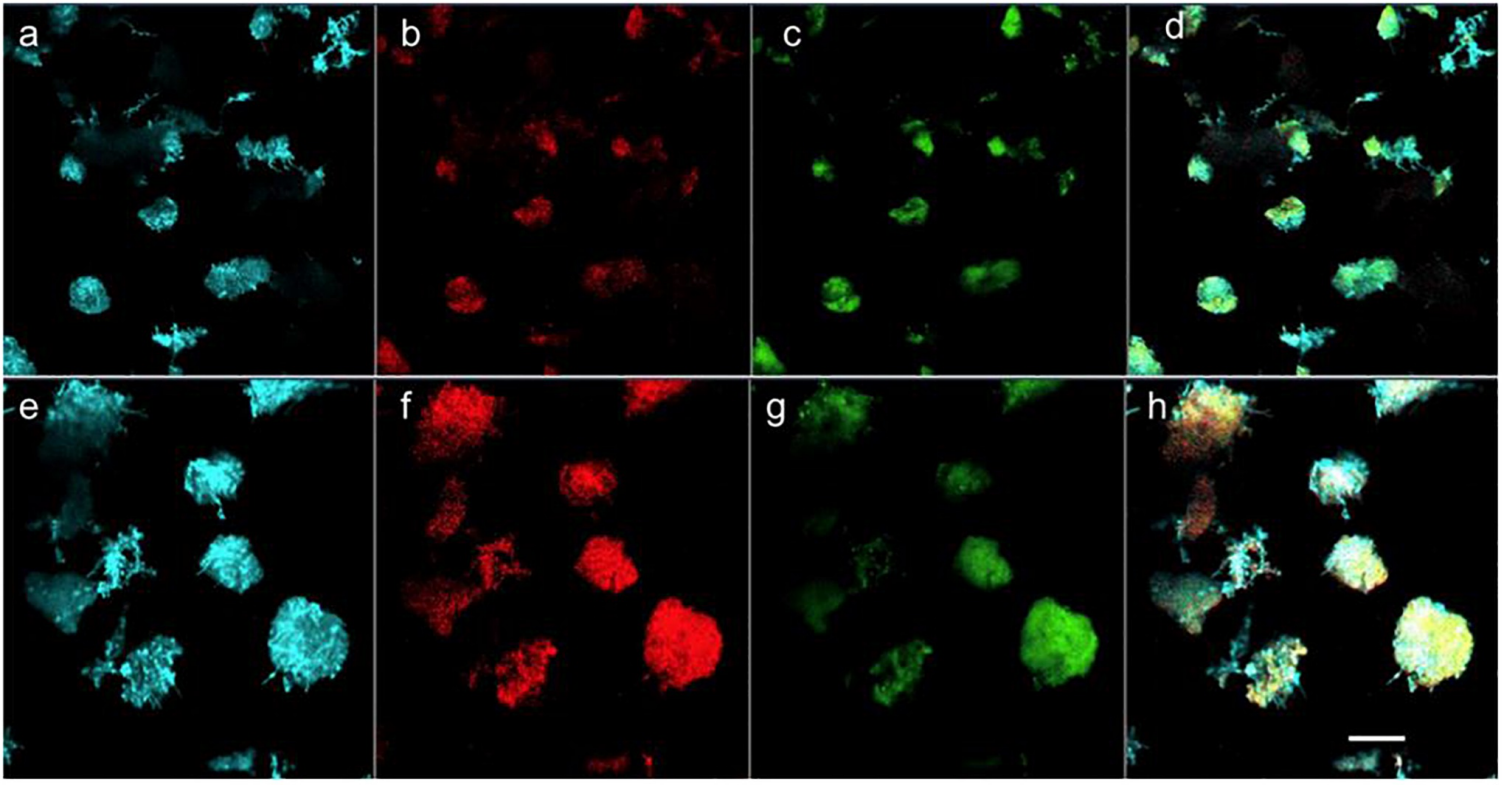
Confocal laser scanning microscopy images of LS IF SBR biomass, illustrating the distribution of EPS in flocs (a, d) before sloughing and, (e-h) following sloughing. Colors correspond to exopolymers with β structures (cyan), α-polysaccharide (red), and protein (green). Scale = 200 μm.

The samples collected from the FS IFAS were probed with molecule-specific fluorescent stains and visualized with CLSM to determine the distribution of EPS and live/dead cells. It was observed that micro-colonies with predominantly dead cells were sloughed from the carrier media (Fig 3). It was observed that before biofilm sloughing, the micro-colonies with protein or polysaccharide-rich extracellular matrix were present in the biofilm. The micro-colonies with predominantly polysaccharide exopolymers were detaching from the biofilm, during sloughing. The concentration of α polysaccharides and polymers with β structures decreased during biofilm sloughing (Fig 4). The large and compact flocs that were rich in α polysaccharides and polymers with β structures were present in the flocs, following biofilm sloughing (Fig 5). The results were consistent with the EPS extraction data, which showed that the protein to polysaccharide ratio of flocs during biofilm sloughing decreased due to an increase in polysaccharide concentration in the extracted EPS.

#### 3.2.2 Protein to polysaccharide ratios in EPS

**Fig 6:**
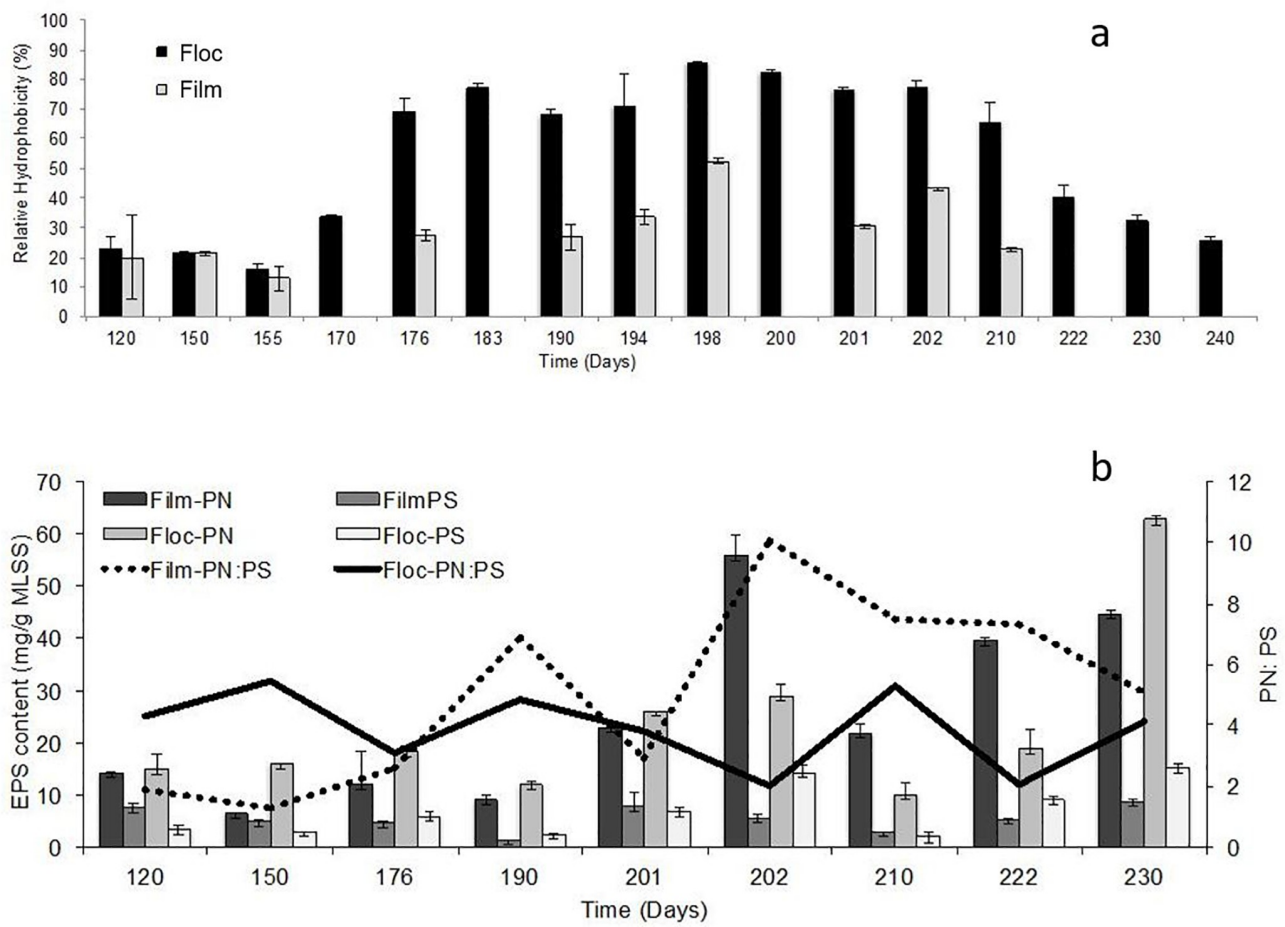
Physical-chemical properties of biofilms and flocs in LS IF-SBR system. (a) Relative hydrophobicity; (b) Composition of EPS.

Proteins and polysaccharides are the major components of EPS. The composition of EPS indicates the structural properties of microbial aggregates. The composition of extracted EPS from biofilm and floc samples of the LS IF–SBR and FS IFAS are presented in Fig 6 and Table 2, respectively. The EPS extraction was performed once or twice a week from the floc samples following the four months of LS IF-SBR operation. The EPS extraction was relatively less often from biofilm samples due to the limited number of carriers media in two liter capacity IF-SBR. EPS was extracted from the biofilm samples when a fluctuation in extracted EPS of flocs or when a sloughing event was observed on the carrier media. The protein to polysaccharide ratio of flocs was more than two-fold higher (28 ±2 mg/g MLSS) compared to biofilms (10 ±2 mg/g MLSS) during the first five months of LS IF-SBR operation (Fig 6). The relative ratio of protein to polysaccharide in biofilms increased (40 ±20 mg/g MLSS) compared to the ratio in flocs (20 ±10 mg/g MLSS) when biofilm sloughing was more recurrent on the carrier media (Figs 1 and 6). The decline in protein to polysaccharide ratio of flocs was associated with higher polysaccharide content. Similarly, in the FS IFAS system, the protein to polysaccharide ratio of the flocs (5.6) was relatively higher compared to the biofilms (4.7) before biofilm sloughing. The protein to polysaccharide ratio was relatively lower in the flocs (1.7) compared to the biofilms (4.3) during sloughing. The decline in the protein to polysaccharide ratio of flocs was due to an increase in polysaccharide content from 18 ±1 to 68 ±2 mg/g MLSS) (Table 2).

#### 3.2.3 Relative hydrophobicity

The relative hydrophobicity of biofilms and flocs was 17–27% during the first five months of LS IF-SBR operation (Fig 6). The relative hydrophobicity of biofilms and flocs gradually increased to 52.5 ±1.1% and 85.8 ±0.2%, respectively, by day 198 (Fig 6). It was observed that the attached biomass on carrier media (biofilm) increased with an increase in relative hydrophobicity of the biomass (Fig 1). The attached biomass on carrier media and relative hydrophobicity of biofilms and flocs decreased following a major sloughing event (Figs 1 and 6).

### 3.3 Microbial community dynamics

**Fig 7:**
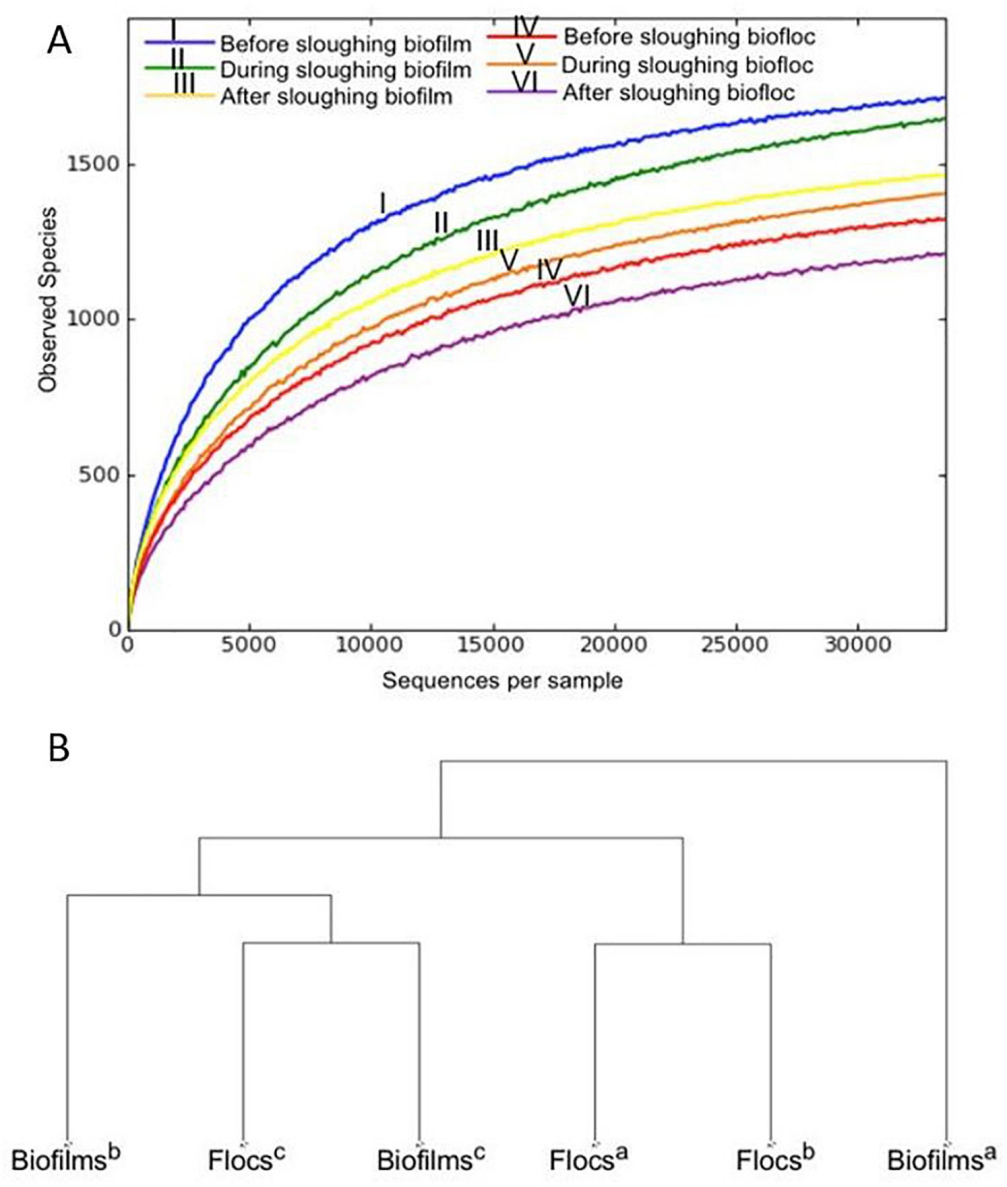
Microbial community diversity in FS IFAS system: (a) α-diversity (using observed species metrics) in biofilms and flocs before, during, and after biofilm sloughing; (b) β-diversity (weighted UniFrac) in biofilms and flocs determined by 16S rRNA gene sequencing, (a) before sloughing, (b) during sloughing and (c) after sloughing.

A total of 270,127 bacterial sequences from Illumina MiSeq were obtained after removing sequences with ambiguous nucleotides and chimeras from all six samples. After assembly and quality filtration, an average of 45,020 bacterial sequences per sample were used for downstream analyses. The rarefaction curves of all the samples for α-diversity were analyzed using observed species (Fig 7) and Shannon Index (S1 Fig). The observed species curves were flatter towards the distal part of the curve (Fig 7). The sequencing depth was enough to reveal the diversity of each sample. Conventionally, replication of samples is considered essential for sequencing to reduce the “jackpot Effect”. However, growing empirical data indicate no significant benefit of triplicate reactions compared to a single reaction for amplicon sequencing [34]. Therefore, a less labor-intensive and economical single reaction protocol was used for amplicon sequencing of the biofilms and flocs of the FS-IFAS system. There are several pros and cons related to 16S rRNA gene sequencing. For example, the DNA-based sequencing methods cannot distinguish between the DNA from live and dead cells, resulting in a biased abundance of the bacterial population in biomass. Illumina sequencing is a semi-quantitative method, and it does not indicate an absolute abundance of bacterial groups. However, 16S rRNA gene sequencing is frequently used because it can provide information on the relative abundance of bacterial groups, identify rare microorganisms in a community, and indicate bacterial groups that may be responsible for changes in species richness in a microbial community [35, 36].

The bacterial community characterization indicated that the microbial population dynamics (in biofilms and flocs) before, during and after biofilm sloughing was very complex. At the bacterial phyla level, the relative abundance of *Proteobacteria* in the biofilm microbial community decreased during biofilm sloughing that recovered after biofilm sloughing. The relative abundance of *Bacteroidetes* before biofilms sloughing was 5.7% that increased during (15.4%) and after (20.4%) biofilm sloughing (S2 Fig). The relative abundance of *Proteobacteria* accounts for 60–72% of microbial communities of the biofilms and flocs. The dynamics of bacterial classes related to *Proteobacteria* in biofilms showed that the relative abundance of *Alphaproteobacteria* was 9.0% that increased during sloughing (10.4%) and decreased after sloughing (8.2%). Conversely, the relative abundance of *Betaproteobacteria* was 43.3% before biofilm sloughing that decreased during sloughing (36.8%) and increased after sloughing (39.0%) (S3 Fig). It indicates that the bacteria related to *Alphaproteobacteria* remain attached to the biofilm or grow during sloughing; whereas, bacteria related to *Betaproteobacteria* are more prone to detachment during sloughing.

**Fig 8:**
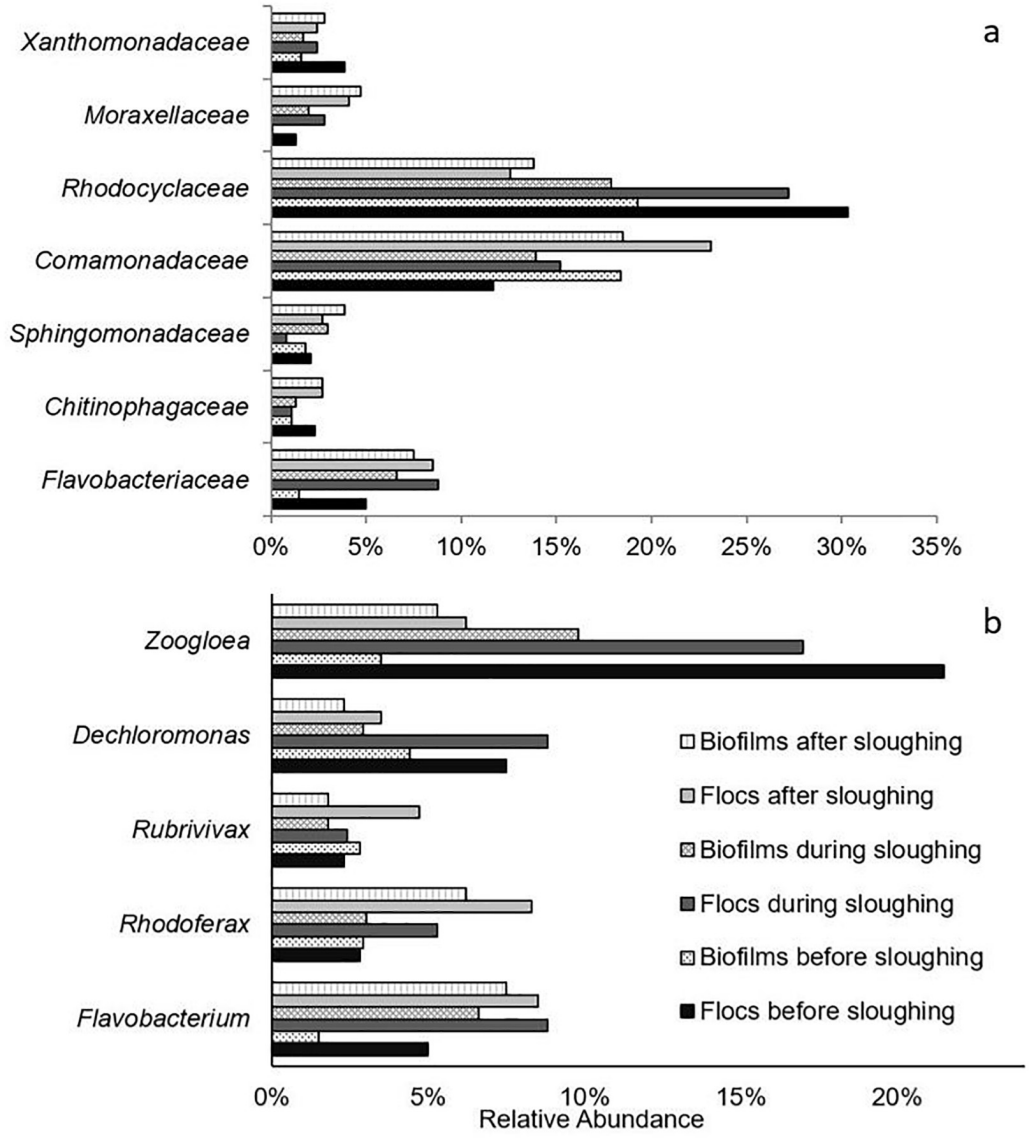
Relative abundance of dominant bacterial groups in biofilms and flocs, before, during, and after biofilm sloughing (a) bacterial families, (b) bacterial genera. X-axis is relative abundance.

Similarly, at the bacterial family level, it was observed that the relative abundance of some bacteria decreased during biofilm sloughing in the microbial community of the biofilms, and its relative abundance in the microbial community of the flocs increased during biofilm sloughing. For example, the relative abundance of *Comamonadaceae* in the microbial community of the biofilms before sloughing was 18.4% that decreased during biofilm sloughing to 13.9%. Conversely, the relative abundance of *Comamonadaceae* in the microbial community of the flocs before sloughing was 11.7% that increased during biofilm sloughing to 15.2% (Fig 8). Furthermore, it was observed that the relative abundance of some bacterial families in the biofilms and flocs decreased during biofilm sloughing (for example, *Rhodocyclaceae*), and the relative abundance of some bacterial families in biofilms and flocs increased during biofilm sloughing (for example, *Flavobacteriaceae*) (Fig 8).

At the genus level, it was observed that the relative abundance of genera *Zoogloea*, *Dechloromonas*, *Rubrivivax*, *Rhodoferax*, and *Flavobacterium* were predominant in microbial communities of the biofilms and flocs in the FS IFAS system (Fig 8). The relative abundance of *Zoogloea* increased in the biofilms and decreased in flocs during biofilm sloughing. Conversely, the relative abundance of *Dechloromonas* decreased in the biofilms and increased in flocs during biofilm sloughing in the FS IFAS system (Fig 8).

## 4 Discussion

Biofilm sloughing was assessed in a laboratory-scale (LS) IF-SBR treating synthetic wastewater and a full-scale (FS) IFAS system treating municipal wastewater. IFAS systems, owing to their hybrid nature, provide an environment where sloughed biofilm can interact with flocs. Biofilm sloughing events resulted in the formation of larger and compact flocs with improved settling properties of suspended biomass (with lower SVI) and higher mixed liquor suspended solids (MLSS). Examination of sloughing in the FS IFAS system revealed changes in the cell viability and composition (Fig 3) and distribution of extracellular polymeric substance (EPS) that may contribute to biofilm dispersion (Fig 4). The 16S rRNA gene sequencing of FS IFAS biofilms and flocs (before, during, and after sloughing) was carried out to understand the population dynamics in the microbial communities of the biofilms and flocs associated with biofilm sloughing.

### 4.1 Biofilm sloughing in IFAS system

In the present study, it was observed that when attached biomass was low, the relative hydrophobicity of the biofilms and flocs was the same in the LS IF-SBR. However, as the attached biomass on carrier media (depth of biofilm) increased, the relative hydrophobicity of flocs was more than two-fold higher compared to the relative hydrophobicity of the biofilms. The relative hydrophobicity is directly proportional to the compactness and better settling properties of microbial aggregates [37, 38]. The gradual increase in the relative hydrophobicity resulted in the formation of compact structures in the LS IF-SBR.

A wider range of floc size was observed, including pinpoint flocs (10%), larger flocs in the range of 50–200 μm (70%), and 20% up to 500 μm in diameter including granular structures (Fig 5). The appearance of granular structures within the reactors suggests that the IF-SBR can generate granular sludge as operated in this study. The operational and nutritional conditions that are required for granulation (feast/ famine conditions, shear force) were present in the LS IF-SBR [18, 39]. Rapid granule formation has been observed in the LS IF-SBR, where the biofilms were relatively thin [20]. However, it is just as likely that in the IF-SBR with mature biofilms, the granular flocs could also have formed as a result of biofilm sloughing with detached biomass associated with suspended floc. In the present study, the ratio SVI_30_/ SVI_10_ of the granular sludge was 0.8 (Table 1) in IF-SBR, indicating granulation in the bioreactors [20, 32, 33]. A small quantity of flocs (up to 35%) is considered beneficial for the stable operations of the granular sludge [40, 41]. The broader range of flocs and granule size in a system harbors more diverse redox properties that result in a more diverse microbial community. The greater microbial diversity and a wider range of flocs and granule size reduce the overall settling properties but increase the stability of the system during granule dispersion and re-granulation [11, 40, 41]. It is possible that carrier media in IF-SBR physically break the larger granules to inhibit the formation of oversized granules and contribute to the co-occurrence flocs with granular sludge.

The results indicate that the sloughing of large particles accounted for the increased MLSS and improved biomass settling properties. The retention of sloughed biomass in the suspended solids is an interesting observation. In the IFAS systems, slow-growing bacteria are relatively enriched on the biofilms compared to the flocs. For example, the denitrifying bacteria in a biofilm’s anoxic zone are relatively slow-growing organisms than the aerobic heterotrophs [7, 42]. In the conventional activated sludge, the slow-growing bacteria are more prone to a washout at low SRT. A recent study has shown that BNR was relatively more efficient in the hybrid bioreactors compared to the biofilms-based bioreactor [10]. It is speculated that retention of sloughed biomass via aggregation with suspended floc is one mechanism that improves the overall biological nutrient removal (BNR) capability in integrated hybrid systems [11, 42].

### 4.2 Mechanism of biofilm sloughing

The polysaccharide content is relatively higher in loosely bound EPS compared to tightly bound EPS [43]. It was observed that the protein to polysaccharide ratio of biofilms and relative hydrophobicity of biofilms increased with biofilm sloughing (Fig 6). Confocal microscopy analyses revealed that polysaccharide rich micro-colonies detached from the biofilms and associated with the flocs (Fig 5). The prevailing view is that polysaccharides support microbial aggregation [15, 44]. Contrarily, polysaccharide-dependent biofilm dispersion has also been reported; however, further studies are required to understand the underlying mechanism [45]. It has been proposed [11] that a stress factor induces the over-expression of polysaccharides that are regulated by quorum sensing [46]. Additionally, downregulation of lectins (polysaccharide binding adhesive proteins) may weaken the microbial aggregation. For example, downregulation of FimH adhesin (mannose-binding adhesin [47] has been observed under phosphorus limiting conditions [48].

A loss of cell viability might have contributed to the weakened cell-to-cell interactions and biofilm sloughing (Fig 3). Biofilm sloughing was predominant when the attached biomass (biofilm depth) was highest during the fifth month of IF-SBR operation (Fig 1). The increasing thickness of biofilms creates nutrient-limiting conditions. A subpopulation of the microbial community lysis when biofilm depth reaches a threshold limit [49, 50]. The distribution of live and dead cells (Fig 3) indicated that dead cells predominated in areas where biofilm sloughing occurred. The internal or external stressors trigger the quorum sensing signals to maintain the biofilm by producing excessive polysaccharides (as described earlier) and endogenous decay. Endogenous decay contributes nutrients to neighboring cells, resulting in a microbial community that is resistant to a stressor to maintain a biofilm. However, when a stressor exceeds a threshold limit, it results in excessive cell lysis and signals the microbial consortium to prepare for dispersal. For example, downregulation of the adhesive pili and upregulation of the flagella facilitates biofilm sloughing [11, 44, 46, 48].

### 4.3 Microbial population dynamics associated with biofilm sloughing

Before biofilm sloughing, the observed species richness (α-diversity) (sampled from the FS IFAS system) was more diverse in biofilms compared to the flocs (Fig 7a). The observed species richness of biofilms decreased, and flocs increased during biofilm sloughing. It indicates that the sloughed micro-colonies contributed to the species richness of flocs. The subsequent decline in diversity of the flocs after biofilm sloughing could be due to the reassociation of microbes with the biofilm [17] or loss from the system (unsettled biomass and/or cell death).

The abundance of bacterial populations was examined through β-diversity analysis using weighted UniFrac metrics [35]. Consistent with the α-diversity analysis described above, the microbial communities of biofilms and flocs were distinct prior to sloughing. Sloughing led to both biofilms and flocs being more similar to each other (Fig 7b). The patterns observed indicate several different changes leading to the changes in relative abundance. For example, differential detachment or sloughing of a subpopulation, the relative decline of microbial groups during sloughing in both fixed-films and suspension, and loss of attached biomass and association with floc. This is reflected in the relative abundance of *Proteobacteria* (phylum), *Bacteroidetes* (phylum), *Betaproteobacteria* (class), *Gammaproteobacteria* (class), *Rhodocyclaceae* (family), and *Flavobacteriaceae* (family) in the biofilms and flocs before (biofilms: 65%, 6%, 43%, 9%,19%, and 2%, respectively; flocs: 72%, 13%, 45%, 7%, 30%, and 5%, respectively) and after sloughing (biofilms: 64%, 20%, 44%, 12%, 14%, and 8%, respectively; flocs: 65%, 20%, 43%, 8%, 13%, and 8%, respectively). Examination of the relative abundance of the residual biofilm remaining on the carrier indicates bacterial groups that were less susceptible to detachment during sloughing. These include the bacterial classes *Alphaproteobacteria*, *Deltaproteobacteria*, *Gammaproteobacteria*, *Cytophagia*, *Flavobacteria*, *Sphingobacteria*, and *Saprospirae* during biofilm sloughing (S2 Fig).

*Comamonadaceae* are heterotrophic bacteria that are known to contribute to denitrification and phosphorous uptake in wastewater treatment [51–54]. The relative abundance of *Comamonadaceae* decreased in biofilms and increased in flocs during sloughing. However, after sloughing, the relative abundance of *Comamonadaceae* increased in biofilms and flocs (Fig 8). It is speculated that the increase in the relative abundance of *Comamonadaceae* on biofilms was due to reattachment of the sloughed biomass and/or regrowth of the bacteria that resulted in the dynamic characteristic of *Comamonadaceae* in the biofilm. The detachment of large and compact particles during sloughing that can support the growth of denitrifying bacteria might have contributed to the subsequent increase in the relative abundance of *Comamonadaceae* in the biofilms and flocs after sloughing in the IFAS system. Further studies are required to understand the dynamic characteristics of these bacteria.

The relative abundance of bacteria related to the family *Flavobacteriaceae* increased more than four-fold in the biofilm during sloughing (Fig 8). It is speculated that the relative abundance of *Flavobacteriaceae* increased because they are present in the core of biofilms and are less prone to sloughing. Additionally, *Flavobacteriaceae* can grow by hydrolysing the soluble microbial products, live and dead cells, and EPS [54]. The relative abundance of bacteria related to the family *Rhodocyclaceae* decreased during and after biofilm sloughing in microbial communities of biofilms and flocs (Fig 8). The bacteria related to the family *Rhodocyclaceae* are known for the extracellular polysaccharides that facilitate microbial adhesion [55]. An increase in the polysaccharide content of the biofilm EPS in relation to protein might have contributed to weakening biofilm interactions and facilitated sloughing [45]. The decline in the relative abundance of bacteria related to family *Rhodocyclaceae* is consistent with CLSM imaging analyses showing that the micro-colonies rich in polysaccharide content were detaching from the biofilms during sloughing. The respective characteristics of the bacterial population (e.g. *Flavobacteriaceae*, *Rhodocyclaceae*, and *Comamonadaceae*) and the dynamics observed in this study indicate slow-growing bacteria and denitrifiers that detach during sloughing events are retained in the system. Retention of the sloughed biomass with flocs in suspension results in the formation of sludge with a wide range of floc and granule size (from pinpoint flocs to granules) in the bioreactor.

Hybrid systems will increasingly play a role in the future of biological wastewater treatment. Interactions between biofilms and flocs is an area warranting further studies. This study illustrates that these interactions are complex and may contribute to greater stability than non-hybrid systems. A mechanism for biomass retention through the formation of compact flocs was observed as was the dynamics of the relative abundance bacterial population that either sloughed-off or were retained and/or reattached on the carrier following sloughing. Future studies through more directed and quantitative molecular approaches [36], such as fluorescent *in situ* hybridization and the composition and distribution of the extracellular protein adhesins (e.g. amyloid adhesins, flagella, pili, and fimbriae) will be valuable in establishing specific roles for different bacterial groups in the changes associated with sloughing in both the biofilm and flocs.

## 5 Conclusion

Biofilm sloughing is a recognized occurrence in biofilm-based bioreactors; however, the fate of sloughed biomass is not well-studied in integrated fixed-film bioreactors. This paper reports on a study of a laboratory-scale IF-SBR treating synthetic wastewater and a full-scale IFAS system treating municipal wastewater. The study demonstrated that sloughed biomass readily associates with the suspended biomass resulting in the formation of large granular flocs and improved settling properties of the suspended biomass. It was observed that microcolonies that were rich in polysaccharide content and dead cells predominantly slough from the biofilms. The α diversity of biofilms decreased and suspended biomass increased during biofilm sloughing, further supporting the interactions between floc and film in hybrid systems resulting from sloughing events. Understanding the fate of sloughed biomass in the IFAS system is of increasing importance given the role of biofilms and hybrid systems in the future of biological wastewater treatment.

## Supporting information

S1 Table Relative intensity of EPS constituents in FS IFAS flocs and biofilms derived from CLSM image analysis. (DOCX)

S1 Fig α-diversity (using Shannon metrics) in biofilms and flocs before, during and after biofilm sloughing. (TIFF)

S2 Fig Relative abundance of dominant bacterial groups in biofilms and flocs, before, during and after biofilm sloughing at bacterial phyla level. X-axis is relative abundance.
(TIFF)

S3 Fig Relative abundance of dominant bacterial groups in biofilms and flocs, before, during and after biofilm sloughing at bacterial classes level. X-axis is relative abundance.
(TIFF)
