# Supplementary material for: Fate of sloughed biomass in integrated fixed-film systems
Source: PLoS One. 2022 Jan 21;17(1):e0262603. doi: 10.1371/journal.pone.0262603 (PMC8782294; doi:10.1371/journal.pone.0262603)
Supplement: S1 Table — (DOCX) [file pone.0262603.s001.docx]

| **S1 Table.** Relative intensity of EPS constituents in FS IFAS flocs and biofilms derived from CLSM image analysis | | |
| --- | --- | --- |
| Fluorescent stain | Target extracellular polymer | Ex/Em^a^ (nm) |
| FITC | Protein | 488/517 |
| Calcofluor White | Cellulose and amyloid protein | 355/433 |
| Concanavalin A^b^ | α-mannopyranosyl and  α-glucopyranosyl residues. | 633/650 |
| ^a^Maximum excitation and emission length  ^b^Concanavalin-A conjugated with Alexa fluor 633 | | |
