# Supplementary figures and images for: Fate of sloughed biomass in integrated fixed-film systems
Source: PLoS One. 2022 Jan 21;17(1):e0262603. doi: 10.1371/journal.pone.0262603 (PMC8782294; doi:10.1371/journal.pone.0262603)

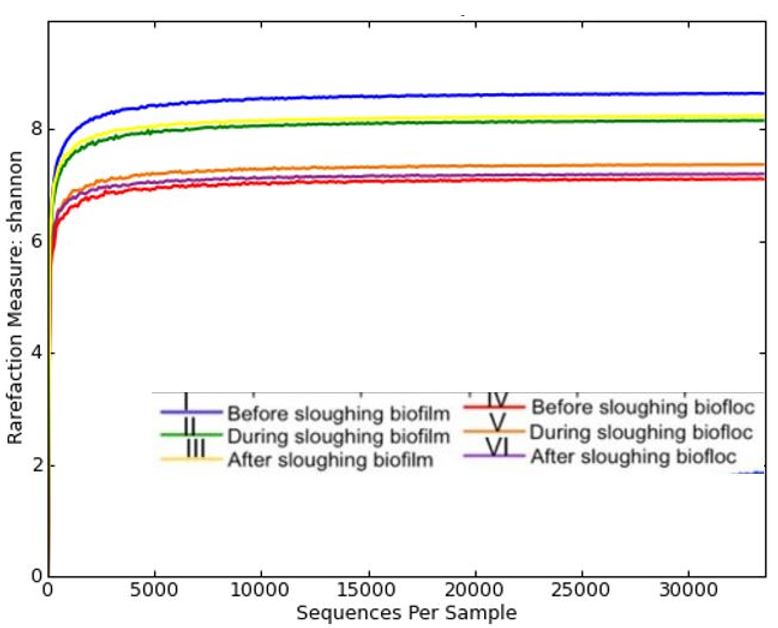

Supplement: S1 Fig — (TIFF) [file pone.0262603.s002.tiff]

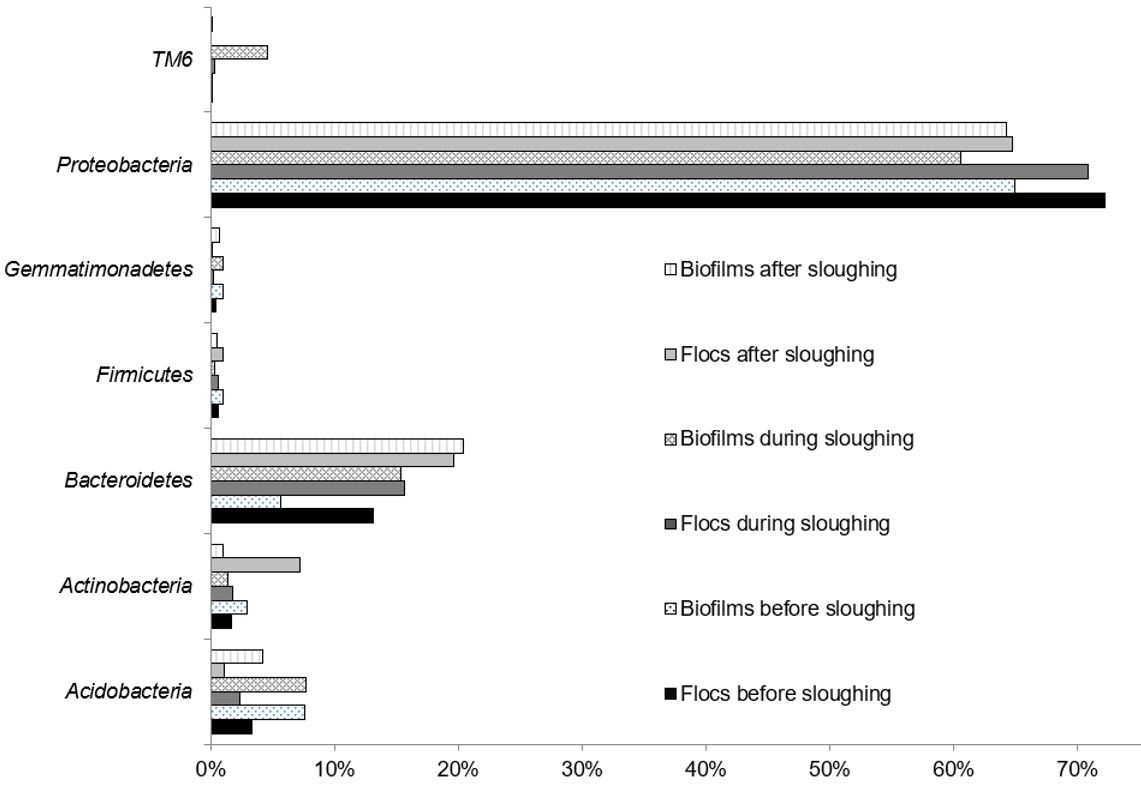

Supplement: S2 Fig — X-axis is relative abundance. (TIFF) [file pone.0262603.s003.tiff]

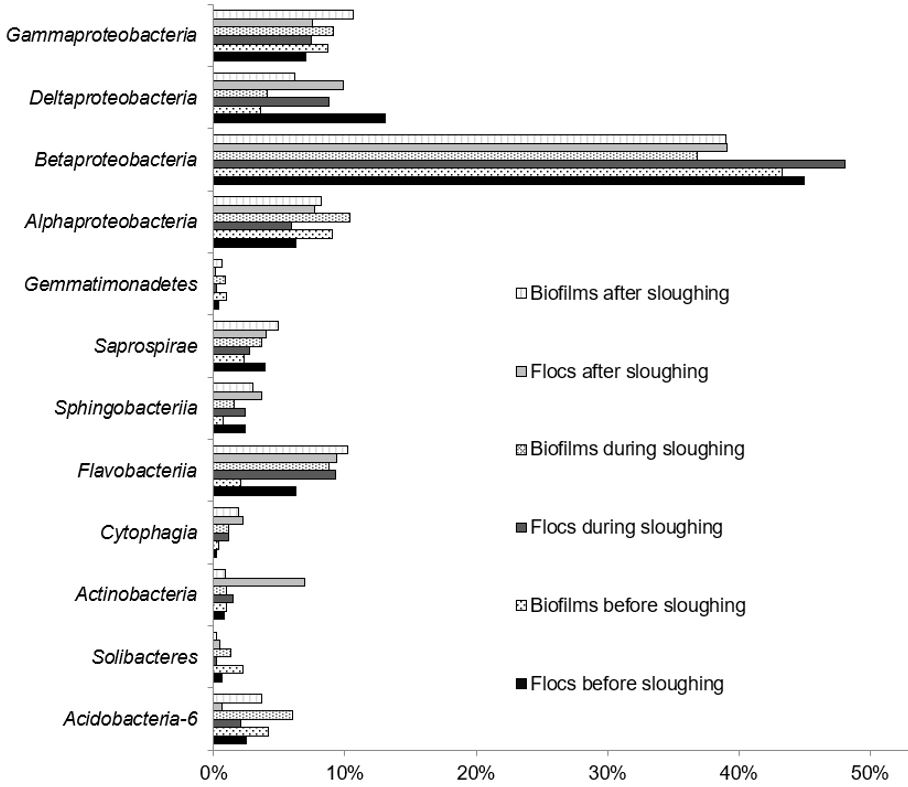

Supplement: S3 Fig — X-axis is relative abundance. (TIFF) [file pone.0262603.s004.tiff]
